# Early-Onset Neutropenia Induced by Rituximab in a Patient With Pemphigus Vulgaris: A Case Report and Literature Review

**DOI:** 10.7759/cureus.110725

**Published:** 2026-06-12

**Authors:** Lamis El Yamani, Ouissal Hormi, Nassiba Zerrouki, Nada Zizi

**Affiliations:** 1 Dermatology, Mohammed VI University Hospital, Oujda, MAR

**Keywords:** biological agents, early-onset neutropenia, mortality, pemphigus vulgaris, rituximab

## Abstract

Rituximab (RTX), a chimeric anti-CD20 monoclonal antibody, is increasingly used in autoimmune diseases. Although generally safe, it may rarely induce cytopenias, particularly early-onset neutropenia (EON) or late-onset neutropenia (LON), with LON being more frequent. We report the case of a 48-year-old female patient with pemphigus vulgaris who developed EON 21 days after RTX initiation. After the second RTX infusion, she presented with severe neutropenia, anemia, fever, respiratory symptoms, and *Staphylococcus aureus* sepsis. Despite antibiotics and intensive care, she died of refractory septic shock.

EON occurs within four weeks of RTX administration and remains less commonly reported than LON. Only 13 cases have been described in the literature, but none was fatal. This is the first reported fatal case of EON in pemphigus vulgaris and the second reported fatal case in a dermatologic disease. This case highlights the need for awareness of EON as a rare but potentially life-threatening RTX complication. Close blood count monitoring during and after RTX therapy may allow earlier detection and intervention.

## Introduction

Biological agents are increasingly used in therapeutic practice, particularly for autoimmune diseases. Rituximab (RTX) is a chimeric anti-CD20 monoclonal antibody targeting the transmembrane surface protein CD20, which is expressed on B cells during their differentiation [1]. First introduced in 1997 for the treatment of non-Hodgkin lymphoma, its indications have progressively expanded to include several autoimmune diseases, especially in rheumatology [2] and more recently in dermatology. Pemphigus vulgaris is a rare, chronic, and potentially life-threatening autoimmune blistering disease affecting the skin and mucous membranes, particularly the oral mucosa. It is caused by pathogenic autoantibodies directed mainly against desmoglein 3 and, in some cases, desmoglein 1. These antibodies disrupt keratinocyte adhesion, leading to acantholysis and the formation of painful erosions or flaccid blisters. Despite systemic corticosteroids and conventional immunosuppressive agents, some patients experience multiple relapses, treatment resistance, or significant therapy-related adverse effects. In this context, RTX has become an important therapeutic option for moderate, severe, or refractory pemphigus vulgaris. By depleting autoreactive B cells, RTX reduces autoantibody production and can induce prolonged clinical remission. However, although RTX is generally considered relatively safe, its increasing use requires better recognition of uncommon but potentially severe complications. The most frequently reported adverse effects include infusion reactions, infections, and severe mucocutaneous reactions. Hematologic complications, particularly cytopenias, have also been described. Two types of neutropenia have been reported after RTX administration: early-onset neutropenia (EON), occurring within the first four weeks after infusion, and late-onset neutropenia (LON), which is more commonly described [2]. In contrast, EON remains rare and probably under-recognized, particularly in dermatologic indications, where systematic blood count monitoring after RTX infusion is not always performed. Because severe neutropenia may rapidly progress to life-threatening infection and sepsis, early identification of this complication is clinically important.

We report this case to highlight a rare but fatal hematologic complication of RTX in a patient treated for pemphigus vulgaris. To the best of our knowledge, this is the first reported fatal case of RTX-associated EON in pemphigus vulgaris. This observation emphasizes the need for increased clinician awareness and supports the importance of close hematologic monitoring during and after RTX therapy.

## Case presentation

**Figure 1:**
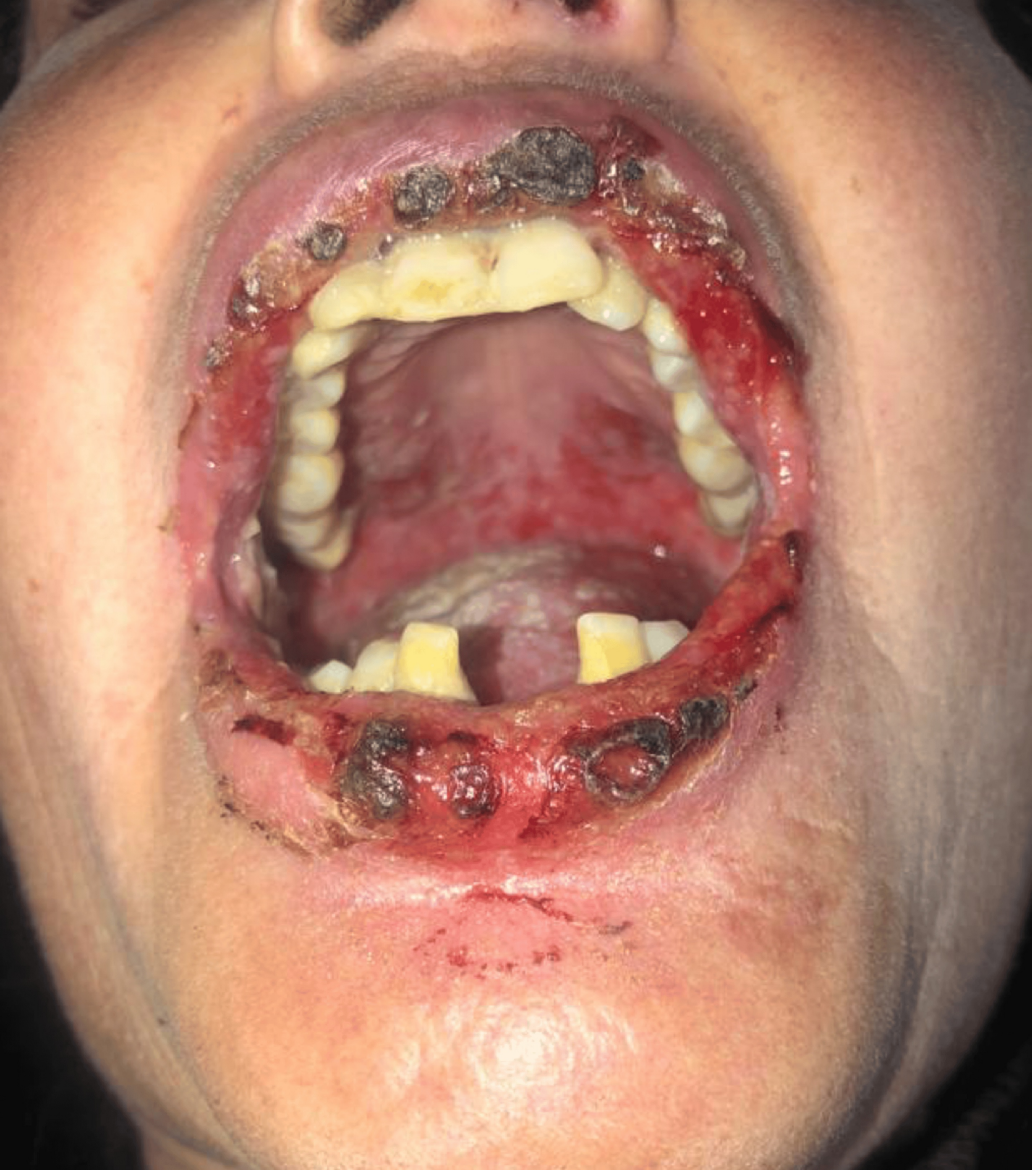
Oral mucosal involvement in pemphigus vulgaris Clinical appearance of oral involvement in pemphigus vulgaris showing multiple painful erosions and ulcerations of the buccal mucosa, associated with hemorrhagic crusts. The lesions result from intraepithelial blistering due to autoantibody-mediated loss of keratinocyte adhesion (acantholysis), leading to fragile bullae that rapidly rupture and leave raw erosive surfaces with bleeding and crust formation.

A 48-year-old female patient had been followed up since 2019 for pemphigus vulgaris (Figure 1), diagnosed based on clinical, histologic, and immunologic criteria (positive anti-desmoglein 1 and 3 antibodies). The patient initially received oral corticosteroid therapy at 2 mg/kg/day in combination with azathioprine 100 mg/day. During corticosteroid tapering, she experienced several relapses while remaining on azathioprine. Azathioprine was continued for three years before being discontinued. RTX therapy was then initiated after azathioprine withdrawal. She received a first infusion of 1,000 mg of RTX, followed by a second infusion of 1,000 mg 22 days later. Prior to the initiation of RTX therapy, a comprehensive pre-treatment evaluation was performed. This included a full clinical assessment and laboratory work-up to rule out active or latent infections. Screening for tuberculosis was negative, including interferon-gamma release assay and chest radiography. Serologic testing for chronic viral infections (hepatitis B virus, hepatitis C virus, and human immunodeficiency virus) showed no evidence of active or prior infection. No biological or clinical signs of systemic infection were identified at baseline. Vaccination status was reviewed before treatment initiation, in accordance with current recommendations for patients planned for B-cell depleting therapy. No contraindications related to vaccination status were identified, and no recent live vaccines had been administered.

Baseline laboratory tests showed a neutrophil count of 10,690/μL, with otherwise normal liver and renal function tests. The evolution of laboratory parameters throughout the clinical course is summarized in Table 1. Twenty-one days after the first RTX infusion, the complete blood count (CBC) revealed a mild decrease in neutrophils to 1,430/μL, along with elevated liver enzymes: aspartate transaminase (AST) 47 U/L, alanine transaminase (ALT) 241 U/L, gamma-glutamyl transferase (GGT) 371 U/L, alkaline phosphatase (ALP) 112 U/L, and C-reactive protein (CRP) 16 mg/L.

**Table 1:** Serial laboratory findings and reference values during the clinical course This table illustrates the progression from initially normal findings to severe neutropenia, systemic inflammation, and sepsis, supporting the diagnosis of RTX-induced early-onset neutropenia complicated by fatal septic shock. ALP, alkaline phosphatase; ALT, alanine transaminase; AST, aspartate transaminase; CRP, C-reactive protein; GGT, gamma-glutamyl transferase; RTX, rituximab

Parameter	Baseline	Day 21 after 1st RTX	Day 1 after 2nd RTX	During infection	Reference values
Neutrophils (cells/μL)	10,690	1,430	80	30	1,500–7,500
Lymphocytes (cells/μL)	2,200	1,500	370	Not reported	1,000–4,000
Hemoglobin (g/dL)	14	13.6	10.8	9.6	12–16
AST (U/L)	46	47	Stable	Stable	10–40
ALT (U/L)	Normal	241	Stable	Stable	7–56
GGT (U/L)	Normal	371	Stable	Stable	9–48
ALP (U/L)	Normal	112	Stable	Stable	44–147
CRP (mg/L)	Normal	16	Stable	108	<5
Procalcitonin (ng/mL)	<0.05	<0.05	<0.05	25	<0.05

However, one day after the second RTX infusion, there was an abrupt drop in neutrophils to 80/μL, lymphopenia at 370/μL, and normocytic normochromic non-regenerative anemia (hemoglobin 10.8 g/dL), with no atypical cells seen on peripheral smear. Liver enzyme values remained stable.

In this context, a broad differential diagnosis was considered, including the following: (i) drug-induced cytopenias, particularly RTX-associated hematologic toxicity; (ii) bone marrow infiltration or an underlying hematologic malignancy, such as acute leukemia or lymphoproliferative disorder; (iii) infection-related bone marrow suppression; (iv) immune-mediated cytopenias, including autoimmune hemolytic anemia and immune neutropenia; and (v) hypersplenism.

Hematologic malignancy was considered unlikely in view of the absence of circulating blasts or atypical cells on peripheral blood smear, together with the very acute onset following RTX administration. Infectious etiology was not supported by clinical presentation or laboratory inflammatory parameters. Immune-mediated cytopenias were also deemed unlikely given the absence of biological evidence of hemolysis. Hypersplenism was considered improbable in the absence of clinical or imaging findings suggestive of splenomegaly. Overall, the temporal relationship with RTX infusion and the systematic exclusion of alternative etiologies supported a diagnosis of RTX-associated acute cytopenias.

According to the WHO-UMC causality assessment system, the association between RTX and EON in our patient was classified as probable/likely. This assessment was based on the strong temporal relationship between RTX administration and the onset of neutropenia, the documented worsening following the second infusion, the absence of concomitant medications known to induce neutropenia, and the absence of evidence of viral infection or other alternative causes.

**Figure 2:**
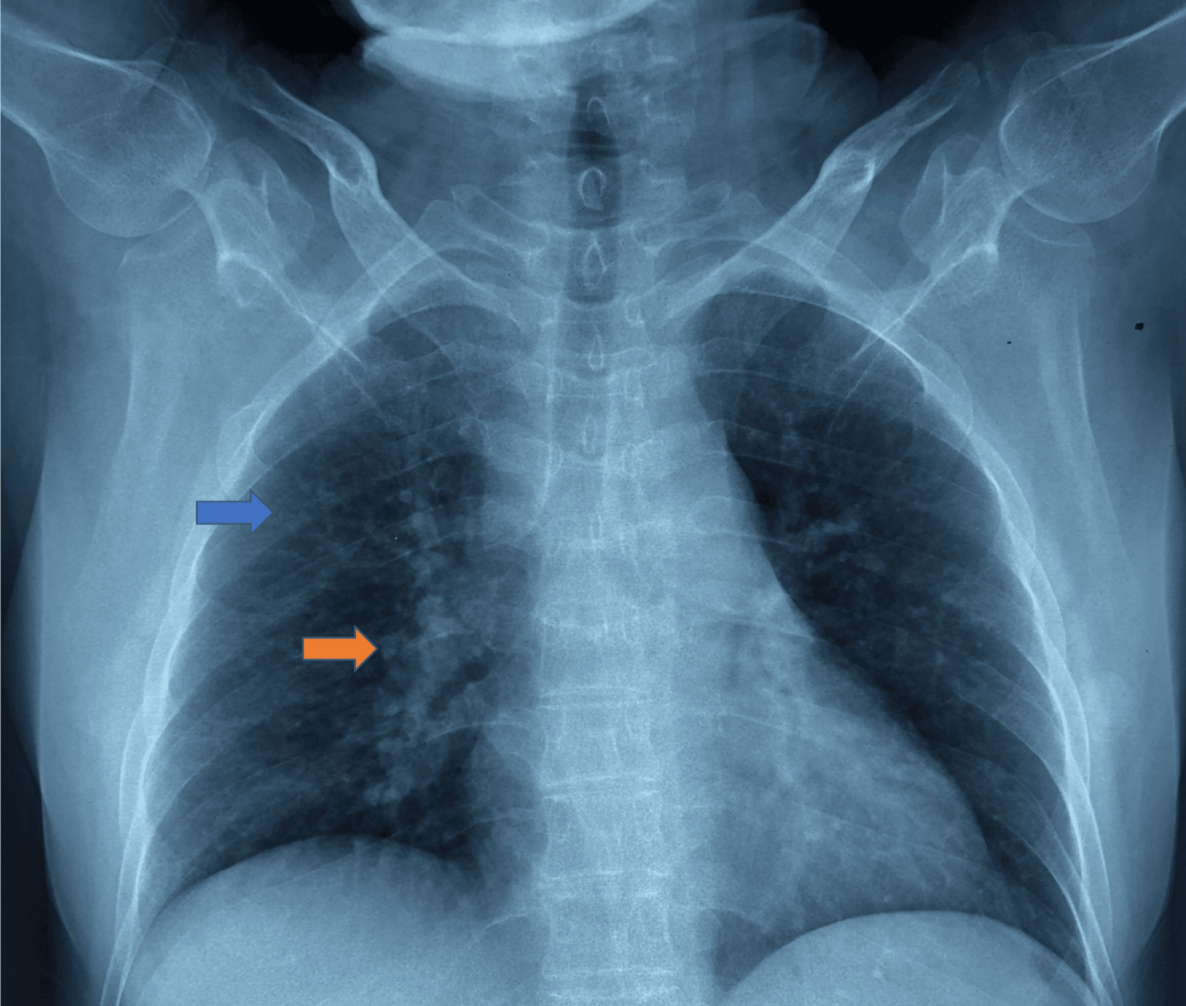
Chest X-ray demonstrating a bronchial and interstitial pattern. The blue arrow indicates bronchial involvement, while the orange arrow highlights interstitial abnormalities.

The following day, the patient developed fever (39°C), chills, and respiratory symptoms. An infectious work-up was performed, including aerobic and anaerobic blood cultures and a urine culture. Blood cultures were positive for *Staphylococcus aureus*, while the urine analysis and culture were unremarkable and showed no evidence of urinary tract infection. The CBC showed a further decline in neutrophils to 30/μL, worsening anemia (9.6 g/dL), CRP 108 mg/L, and procalcitonin 25 ng/mL. Liver enzymes remained unchanged. Chest X-ray showed a bronchial and interstitial pattern, more pronounced on the right side (Figure 2).

The patient was immediately placed in isolation, and empirical broad-spectrum intravenous antibiotic therapy was initiated. A diagnosis of RTX-induced EON was made. However, her condition deteriorated rapidly within a few hours, with the onset of agitation, fever (39.2°C), tachycardia (128 bpm), and hypotension (blood pressure 82/45 mmHg; mean arterial pressure 57 mmHg). Urine output decreased progressively to less than 0.3 mL/kg/h, indicating poor organ perfusion. Initial fluid resuscitation with 30 mL/kg of intravenous crystalloids was administered, followed by norepinephrine infusion due to persistent hypotension. Despite these measures, the mean arterial pressure remained below 65 mmHg. Laboratory investigations showed elevated serum lactate levels (5.8 mmol/L), worsening inflammatory markers, and acute kidney injury (serum creatinine increased from 0.9 to 1.8 mg/dL). Blood cultures were positive for *Staphylococcus aureus*. The patient subsequently developed refractory septic shock with progressive multiorgan failure and suffered a cardiac arrest. Resuscitation efforts were unsuccessful, and she died before granulocyte colony-stimulating factor (G-CSF) could be considered.

## Discussion

RTX is currently considered a first-line treatment for moderate-to-severe pemphigus, especially pemphigus vulgaris and pemphigus foliaceus. By depleting B cells, RTX leads to a marked reduction in circulating anti-desmoglein autoantibodies and significant improvement in lesions. The U.S. Food and Drug Administration approved its use in the treatment of pemphigus vulgaris in June 2018, based on a randomized controlled trial of 90 patients conducted in France comparing RTX plus prednisone with high-dose prednisone alone. This study, the only known controlled trial of RTX in pemphigus vulgaris, showed that 89% of patients receiving RTX achieved remission versus 34% of those treated with prednisone alone, with a corticosteroid-sparing effect [3-5].

The most common adverse effects reported after RTX therapy are infusion reactions. Infections have also been reported, although the risk remains uncertain due to the concomitant use of other immunosuppressive agents alongside RTX. Other reported events include deep vein thrombosis, pulmonary embolism, and several cases of progressive multifocal leukoencephalopathy [6].

RTX-associated neutropenia is an uncommon adverse event, with reported incidences ranging from approximately 0.02% to 6.6% [7]. LON occurs more than four weeks after RTX administration, whereas EON develops within the first four weeks. Most cases correspond to LON, while EON remains much rarer and is mainly described in isolated case reports [7]. In RTX-treated non-Hodgkin lymphoma patients, the reported incidence of LON was 8% [1]. Thrombocytopenia has been described in several case reports in association with EON. The pathogenesis underlying EON remains unclear, and more data are available for LON due to its higher frequency. Since neutrophils and platelets do not express CD20 on their surface, a direct drug-induced lysis similar to B-cell depletion cannot explain the phenomenon [8]. Lin et al. suggested that EON may result from the release of cytotoxic mediators, such as lysozyme and granzyme, leading to collateral neutrophil injury. Genetic susceptibility may also play a role, as patients carrying the high-affinity FcγRIIIa 158V variant could experience more intense immune-mediated B-cell clearance and, consequently, a deeper neutrophil decline. Although FcγRIIIa genotyping was not performed in our patient, this mechanism may be relevant given the abrupt onset and profound severity of neutropenia observed shortly after RTX administration [9].

A literature review was conducted to identify previously reported cases of RTX-associated EON. PubMed/MEDLINE, Google Scholar, and Scopus were searched from database inception to 2025 using the following keywords: “rituximab”, “early-onset neutropenia”, “early onset neutropenia”, “rituximab-induced neutropenia”, “rituximab-associated neutropenia”, “agranulocytosis”, “cytopenia”. Only cases in which neutropenia occurred within four weeks after RTX administration were included. Cases of late-onset neutropenia, defined as neutropenia occurring more than four weeks after RTX exposure, were excluded.

To our knowledge, 13 cases of RTX-associated EON have been reported to date. These included five cases in patients with systemic lupus erythematosus or lupus nephritis [2,3,10,11], two cases in patients with neuromyelitis optica [12], four cases in patients with rheumatoid arthritis [9], one case in a patient with mantle cell lymphoma [7], and one case in a patient with bullous pemphigoid [13]. Most patients were women, as in our observation. The onset of EON ranged from as early as three days to as late as 28 days after RTX administration. RTX was introduced as monotherapy in only seven patients, while the others received concomitant treatments. Resolution was spontaneous in seven patients, and six patients responded positively to granulocyte colony-stimulating factor (G-CSF). No fatal outcomes have been reported to date. The main characteristics of these cases are summarized in Table 2.

**Table 2:** Reported cases of early-onset neutropenia after RTX. ANA, antinuclear antibody; ANC, absolute neutrophil count; AQP4, aquaporin-4; BP, bullous pemphigoid; CS, corticosteroids; EON, early-onset neutropenia; G-CSF, granulocyte colony-stimulating factor; IV, intravenous; MMF, mycophenolate mofetil; NMO, neuromyelitis optica; NR, not reported; RTX, rituximab; SC, subcutaneous; SLE, systemic lupus erythematosus; TMA, thrombotic microangiopathy

Reference	Age/sex	Ethnicity	Disease/indication	Active clinical manifestations	Prior immunosuppressive therapy	RTX regimen	Concomitant treatment	Time to EON	Lowest ANC	Clinical presentation	G-CSF	Outcome
Gottenberg et al., 2005 [10]	22/F	NR	SLE	Articular manifestations	Not reported	375 mg/m²	Corticosteroids, mycophenolate mofetil	15 days after the 1st RTX dose	0.7 × 10⁹/L	EON	Not reported	Resolved spontaneously
Gottenberg et al., 2005 [10]	30/F	NR	SLE	Pleuropericarditis	Not reported	375 mg/m²	None	10 days after the 1st RTX dose	0.6 × 10⁹/L	EON	Not reported	Not reported
Enríquez et al., 2007 [11]	48/F	White	SLE / lupus nephritis	Proliferative lupus nephritis	Cyclophosphamide	375 mg/m²	Not reported	15 days after the 1st RTX dose	0.85 × 10⁹/L	EON	Yes; filgrastim	Resolved after filgrastim
Arroyo-Ávila et al., 2015 [3]	32/F	Hispanic	SLE / lupus nephritis + hemolytic anemia	Proliferative lupus nephritis and hemolytic anemia	Cyclophosphamide	375 mg/m²	Corticosteroids, mycophenolate mofetil	15 days after the 1st RTX dose	0.3 × 10⁹/L	EON + thrombocytopenia	Not reported	Resolved spontaneously
Mealy and Levy, 2015 — Case 1 [12]	32/F	White	Neuromyelitis optica	Longitudinally extensive transverse myelitis, optic neuritis, anti-AQP4 positivity	Not reported	1000 mg	None	7 days after RTX infusion	0.0 × 10⁹/L	Symptomatic agranulocytosis	Yes; filgrastim	Resolved after filgrastim
Mealy and Levy, 2015 — Case 2 [12]	32/F	White	Neuromyelitis optica	Longitudinally extensive transverse myelitis, anti-AQP4 positivity	Not reported	1000 mg/m²	None	28 days after the 1st RTX dose	0.4 × 10⁹/L	Symptomatic agranulocytosis	Yes; filgrastim	Resolved after filgrastim
Shah et al., 2019 [2]	35/F	South Indian	Refractory lupus nephritis	Fever, alopecia, malar rash, photosensitivity, pedal edema progressing to anasarca; class IV proliferative lupus nephritis with thrombotic microangiopathy	Cyclophosphamide	1 g ×2 doses, 14 days apart	Prednisolone, hydroxychloroquine, enalapril; broad-spectrum antibiotics during cytopenia	3 days after the 1st RTX infusion; recurrence after 2nd dose	118 cells/mm³ after 1st dose; 243 cells/mm³ after 2nd dose	Asymptomatic EON + thrombocytopenia	Yes; G-CSF 300 µg SC daily	Neutropenia recovered with G-CSF; platelets recovered spontaneously; patient doing well
Adler et al., 2019 [13]	46/F	East Asian	Bullous pemphigoid	Refractory oral involvement of bullous pemphigoid	Prednisone, mycophenolate mofetil, doxycycline, niacinamide	375 mg/m² weekly ×4; second cycle started 4 months later	Corticosteroids, mycophenolate mofetil	18 days after the 1st RTX dose	0.0 × 10⁹/L	Neutropenic fever with respiratory symptoms	Yes; filgrastim 300 µg SC ×2 doses	Resolved after filgrastim
Lin et al., 2020 [9]	Mean 58 years; 3F/1M: 65F, 33M, 65F, 69F	Not reported	Rheumatoid arthritis	Not reported	First RTX cycle in two patients; 2–3 previous RTX cycles in two patients	1 g ×2 doses, 2 weeks apart, with 100 mg IV methylprednisolone	Methotrexate 15 mg in one patient; hydroxychloroquine 200 mg in one patient	4, 11, and 14 days after RTX infusion; mean 10 days	Mean nadir 0.57 × 10⁹/L; range 0.05–1.17 × 10⁹/L	One neutropenic sepsis; three uncomplicated cases	Yes in one case; no in three cases	Recovery; one case treated with standard protocol + G-CSF; three resolved spontaneously
Nelson et al., 2021 [7]	65/M	Not reported	High-risk stage IV mantle cell lymphoma involving bone marrow and right pleural effusion	Not reported	Bendamustine + RTX initially; later ibrutinib monotherapy for 5 months	RTX 375 mg/m², first dose	Ibrutinib	6 days after the 1st RTX infusion	ANC <0.03 × 10³/µL	Neutropenic fever, temperature 38.3°C; cultures and infectious work-up negative	Yes; G-CSF + broad-spectrum antibiotics	Recovery within 5 days; ANC improved to 4.12 × 10³/µL; afebrile; ibrutinib restarted without further neutropenia
Present case	48/F	Not reported	Pemphigus	Pemphigus vulgaris	Azathioprine	1 g, two doses 22 days apart	None	21 days after the 1st infusion	30/µL	*Staphylococcus aureus* bacteremia, septic shock	No, death before G-CSF	Death

The earliest reported cases of RTX-induced EON were described in patients with systemic lupus erythematosus. Gottenberg et al. reported two women who developed neutropenia 10 and 15 days after the first RTX infusion. Both had SLE-related manifestations, including pleuropericarditis in one case and articular involvement in the other. One case resolved spontaneously, while the outcome was not fully documented in the second [10]. Enríquez et al. reported EON in a 48-year-old female patient treated with RTX for proliferative lupus nephritis. Neutropenia occurred 15 days after the first infusion, following previous exposure to cyclophosphamide. The case raised the possibility that prior cytotoxic or immunosuppressive therapy may contribute to hematologic vulnerability [11]. Arroyo-Ávila et al. described the case of a 32-year-old female patient with proliferative lupus nephritis and hemolytic anemia who developed severe EON after RTX therapy. Neutropenia appeared 15 days after the first infusion and was associated with thrombocytopenia. The cytopenias resolved spontaneously, supporting a possible RTX-related mechanism [3]. Mealy and Levy reported two cases of symptomatic early-onset agranulocytosis in patients with neuromyelitis optica treated with RTX. Both patients developed fever and infectious symptoms approximately two weeks after RTX exposure, with severe neutropenia. They improved rapidly after treatment with filgrastim, allowing continuation of RTX without recurrence [12]. Shah et al. described the case of a female patient with refractory lupus nephritis who developed leukopenia, severe neutropenia, and thrombocytopenia after RTX. The cytopenias improved after G-CSF administration, but recurred after a second RTX infusion, strongly supporting a causal association. The patient ultimately recovered with G-CSF support and close monitoring [2]. Adler et al. described EON in a patient with refractory bullous pemphigoid treated with RTX. Severe neutropenia occurred 18 days after the first infusion and was complicated by fever and respiratory symptoms. The patient improved with antibiotics and filgrastim, although concomitant mycophenolate mofetil and corticosteroids may have contributed [13]. Lin et al. reported four cases of EON in patients with rheumatoid arthritis receiving RTX. Neutropenia occurred between 4 and 14 days after infusion. One patient developed neutropenic sepsis requiring antibiotics and G-CSF, while the others recovered spontaneously. This series emphasized the importance of blood count monitoring after RTX [9]. Nelson et al. described EON in a patient with mantle cell lymphoma who developed severe neutropenic fever after RTX and bendamustine. Despite the presence of another myelotoxic agent, RTX was considered a possible contributor, particularly because bendamustine had previously been tolerated. The patient recovered after G-CSF and antibiotic therapy [7].

Overall, these cases indicate that RTX-induced EON can occur in various autoimmune and hematological diseases. The onset is usually rapid, ranging from 3 to 28 days after RTX exposure. Clinical severity varies from asymptomatic neutropenia to agranulocytosis, neutropenic fever, sepsis, or multilineage cytopenias. Most patients recovered with close monitoring, antibiotics when needed, and G-CSF in severe cases. Compared with previously published cases, the present case is remarkable for its fatal outcome. Our patient developed profound neutropenia after RTX administered for pemphigus vulgaris, complicated by *Staphylococcus aureus* bacteremia and septic shock, with death occurring before G-CSF could be administered. While most reported cases of EON recovered, this case demonstrates that early RTX-associated neutropenia should not be regarded as uniformly benign. Early CBC monitoring after RTX infusion, prompt recognition of neutropenia, rapid infectious evaluation, and immediate initiation of broad-spectrum antibiotics and G-CSF when clinically indicated may be crucial to prevent severe or fatal complications. We are the first to report a case of RTX-induced EON in a patient with pemphigus vulgaris with a fatal outcome. This is also the second reported case of EON due to RTX in the context of a dermatologic disease. However, some hypotheses suggest immune-mediated lysis via circulating CD20 antigens to explain RTX-associated neutropenia, while others propose excessive B lymphopoiesis induced by elevated B-cell activating factor (BAFF) levels, which may inhibit neutrophil production [7].

This report has some important limitations. As it describes only one patient, the association between RTX and EON cannot be confirmed with certainty. Moreover, the patient had severe pemphigus vulgaris and had been exposed to previous immunosuppressive treatments, which may have increased both hematologic fragility and susceptibility to infection. In addition, EON related to RTX is still rarely described in the literature; therefore, its exact frequency, predisposing factors, and best monitoring approach remain unclear.

## Conclusions

RTX-induced EON, although rare, is a potentially serious and even fatal adverse effect, as illustrated by our case. This observation highlights the importance of close hematologic monitoring in the weeks following RTX administration, even in the absence of identified risk factors. As the use of this biologic agent expands to numerous dermatologic conditions, it is imperative that clinicians remain vigilant regarding this underrecognized complication.
